# Supervoxel Segmentation with Voxel-Related Gaussian Mixture Model

**DOI:** 10.3390/s18010128

**Published:** 2018-01-05

**Authors:** Zhihua Ban, Zhong Chen, Jianguo Liu

**Affiliations:** National Key Laboratory of Science and Technology on Multi-spectral Information Processing, School of Automation, Huazhong University of Science and Technology, Wuhan 430074, China; jgliu@ieee.org

**Keywords:** superpixel, supervoxel, video segmentation, Gaussian mixture model, expectation– maximization

## Abstract

Extended from superpixel segmentation by adding an additional constraint on temporal consistency, supervoxel segmentation is to partition video frames into atomic segments. In this work, we propose a novel scheme for supervoxel segmentation to address the problem of new and moving objects, where the segmentation is performed on every two consecutive frames and thus each internal frame has two valid superpixel segmentations. This scheme provides coarse-grained parallel ability, and subsequent algorithms can validate their result using two segmentations that will further improve robustness. To implement this scheme, a voxel-related Gaussian mixture model (GMM) is proposed, in which each supervoxel is assumed to be distributed in a local region and represented by two Gaussian distributions that share the same color parameters to capture temporal consistency. Our algorithm has a lower complexity with respect to frame size than the traditional GMM. According to our experiments, it also outperforms the state-of-the-art in accuracy.

## 1. Introduction

Superpixel segmentation is to partition a still image into atomic segments of similar size and adhering to object boundaries, namely superpixels [1,2,3,4]. In recent decades, superpixel segmentation has been found to be a very useful preprocessing step in many computer vision tasks (e.g., object detection [5,6,7], image segmentation [8,9,10], visual saliency [11], and noise estimation [12]). This is mainly because superpixels improve the computational efficiency and robustness of subsequent applications by reducing the number of inputs and removing a large amount of redundant information.

Because of the effectiveness of superpixel segmentation, the idea of partitioning data points into homogeneous atomic clusters has been extended into video analysis by adding a constraint on temporal consistency [13,14]. The new atomic cluster in video is called the *supervoxel*, as the video analog to the superpixel in a still image [15]. In addition to the constraints inherited from superpixel segmentation (i.e., adhering to object boundaries and having similar size), the new temporal constraint—namely spatiotemporal coherence—requires that a supervoxel belong to the same object over time. Superpixel and supervoxel are used interchangeably in the following text because a supervoxel in a single frame is a superpixel. Existing works solve the supervoxel problem by either stacking video frames together as 3D volumetric data and performing segmentation by treating the time axis as an additional spatial dimension (e.g., [1,16]), or tracking or propagating the initial superpixel segmentation from the first frame through inferring temporal correspondence in successive frames (e.g., [17,18,19]). Methods falling into the first category cluster video pixels in 3D Euclidean space with color information added to each point. This strategy may result in supervoxels only preserving temporal consistency in very few neighboring frames. When more frames are considered, the same object in the video can be easily separated into different supervoxels, even if the video is completely stacked by the same single still image. These kinds of methods seem to be more suitable for real 3D volumetric data (e.g., 3D electron microscope (EM) images), and not appropriate for video.

**Figure 1:**
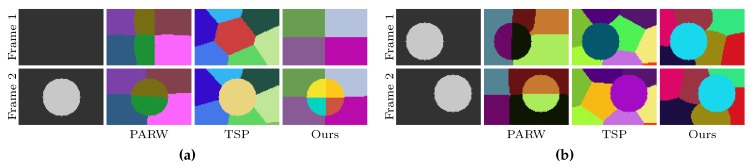
Two toy examples of supervoxel segmentation on (**a**) video with new object and (**b**) video with moving object. The first row are synthetic video frames. The results of two representative state-of-the-art algorithms—PARW [17] and TSP [20]—are plotted in the second and third rows. Our results are shown in the last row. Pixels with the same supervoxel label are painted using the same color, best viewed in color.

More methods explore the second strategy to improve the temporal consistency of supervoxels. Generally, those methods extract the superpixels of the current frame by using the immutable segmentation of the previous frames [13,17,20]. For instance, the method using partially absorbing random walks (PARW) [17] initializes superpixels for the current frame using the seeds of the previous frame and generates new superpixels based on the current frame and the next frame. Because the segmentations of the previous frames are immutable, the seeds that were used to initialize superpixels of the current frame may change to a different object due to occlusions, and thus the temporal consistency is easily lost (Figure 1a illustrates this problem). As shown in Figure 1a, temporal superpixels (TSP) [20] is able to detect “dead” and “new” superpixels to deal with object occlusion. However, their model overacts when dealing with moving objects, as shown in Figure 1b. Moreover, most of the existing supervoxel algorithms rely on optical flows [18,19,21], which strongly influence their temporal consistency and execution speed.

**Figure 2:**
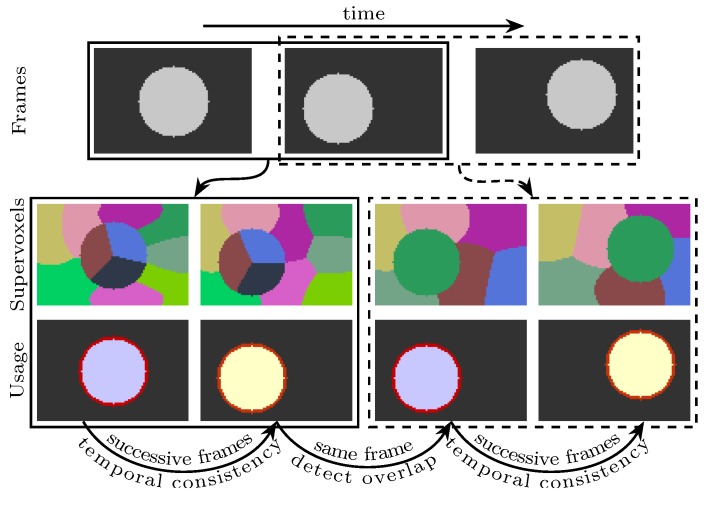
An illustration of a possible usage of the proposed method. The first row is three successive frames. In the second row, the first two superpixel segmentations are extracted using only the first two frames. Similarly, the last two superpixel segmentations are extracted using only the last two frames. In this example, the second frame has two superpixel segmentations. The third row explains a possible usage in foreground segmentation or object tracking, best viewed in color.

Aiming to improve the temporal consistency of supervoxel segmentation when video is with object occlusion and moving objects, we propose an alternative scheme that takes only two adjacent frames into account at a time and produces two valid segmentations for each internal frame. Except for the original colors and spatial locations, our method does not rely on any precomputed information (e.g. optical flows). This scheme has two major benefits: (a) it provides a coarse-grained parallelism that every two frames can be segmented in parallel; (b) each internal frame having two segmentations gives the subsequent applications an opportunity to validate their results. A possible usage of our supervoxels is depicted in Figure 2, where the propagation of a segmentation is not performed at the superpixel level but at the object level.

The traditional Gaussian mixture model (GMM)—a weighted sum of Gaussian functions—has been widely applied to the problem of classification [22,23]. However, it cannot be directly applied to supervoxel segmentation because its computational complexity is relatively high and it does not encode the constraint on segment size. GMM has been explored for superpixel segmentation and achieved good segmentation accuracy in our previous work [24]; however, temporal consistency was not considered. In this work, we extend the model of [24] to supervoxel segmentation and propose a voxel-related GMM to tackle temporal consistency. Inherited from [24], we use constant weights and subsets of all the Gaussian functions in the sums to ensure that the produced segments have similar sizes and that the algorithm has linear complexity. In our new model, the size of the subsets is controllable so that we can tune it to track objects with different moving speed. For instance, a large size is suitable for videos containing fast-moving objects (see Section 3.4 for more details). Each supervoxel is composed of two superpixels on two consecutive frames, and each superpixel is represented by a Gaussian distribution. To ensure temporal consistency, we use the same color parameters (i.e., color mean vector and color covariance matrix) for the two distributions of each supervoxel. This is mainly because the same object in two consecutive frames tends to be similar in color. Experiments conducted on a well-known dataset show that the proposed algorithm is superior to the state-of-the-art in terms of accuracy.

The rest of this paper is organized as follows: some related works are introduced in Section 2. Section 3 presents our supervoxel method. Experiments are conducted in Section 4. Finally, we conclude our work in Section 5.

## 2. Related Works

Using superpixels as basic elements for image analysis and processing was first introduced by Ren and Malik [25]. After the algorithms proposed in [1,16,26], the following works of extracting superpixels for video data began to take temporal consistency into account. In this section, we will review some representative algorithms that are related to supervoxel segmentation.

The hierarchical graph-based method (GBH) [26] constructs a 3D graph using all the video frames and applies Felzenszwalb and Huttenlocher’s algorithm [27] to iteratively merge voxels using a hierarchical scheme. Instead of building a regular grid graph base on a 26-neighborhood in 3D spatial temporal space, the edges between frames are built in such a way that each voxel is connected to its nine neighbors along the backward flow vector. The flow vectors are also used in the clustering process. This method uses dense optical flow to ensure temporal consistency. However, errors from the precomputed flows may shift to the segmentation process. Additionally, GBH cannot generate supervoxels with similar size, and the number of supervoxels cannot be directly controlled. Since GBH requires all frames to be loaded into memory (in which case it may fail for a longer video), Ref. [28] gives an implementation of GBH to make it have a streaming capability by using a Markov assumption.

Simple linear iterative clustering (SLIC) [1] uses a modified *k*-means to group pixels based on their spatial location in a still image. In each iteration of SLIC, the search space of the current superpixel is limited to a square region whose center is the spatial center of the current superpixel and whose size is proportional to the desired size of each superpixel. To extend the modified *k*-means to video data, voxels in frames are seen as points in 3D Euclidean space, and thus the search space becomes a cube. Seeds are regularly distributed among the fake 3D volumetric data, and as a result supervoxels are temporally consistent only over a short range of frames. In SLIC, small connected regions are merged into neighboring supervoxels in a 3D 10-neighborhood. However, a moving object captured in different frames may not be connected in the 10-neighborhood. Therefore, the merging step may cause a negative effect on the segmentation accuracy. Similar to SLIC, the method besed on graph cuts (GC) [16] also stacks all frames together as a fake 3D volume. GC extracts supervoxels by partitioning graphs in an energy minimization framework optimized using graph cuts. However, GC cannot guarantee temporal consistency for a long range of frames.

Spatiotemporal closure (SC) [18] starts by extracting superpixels in the first frame using the original method of TurboPixels [3]. The seeds for the superpixel segmentation of the next frame are projected along the weighted flow vectors from the segmented seeds of the current frame. This method also relies on precomputed optical flow and is not self-contained. An incorrect flow vector may easily produce a supervoxel that covers multiple objects. Following works like Ref. [13], PARW [17] use a similar method to move seeds from the superpixel segmentation of the current frame to the next adjacent frame. Although TSP [20] does not move seeds with the aid of optical flow, seeds are still moved from an immutable superpixel segmentation, and the seeds may be further evolved to a new object. Superpixels Extracted via Energy-Driven Sampling (SEEDS) [29,30] was first designed for superpixel extraction, and is extended to video data in video SEEDS (vSEEDS)[19]. Instead of moving superpixel representatives from previous segmentations, vSEEDS propagates the rough block-level segmentation of each frame into the next frame. However, vSEEDS still shares the same drawback with the seeds moving methods when dealing with new objects.

Overall, existing supervoxel algorithms generally require motion information to aid the segmentation. However, this kind of information is not originally equipped with the frames, and needs to be computed by additional algorithms. The error in motion vectors may result in erroneous results in supervoxels. Algorithms that evolve previous superpixel segmentation to new frames usually make the previous segmentation immutable. Because the previous labels may shift to a new object, these methods often fail to handle new objects or moving objects.

## 3. The Method

In the proposed method, the supervoxel problem is simplified as supervoxel segmentation on two adjacent frames (see Figure 2 for an illustration). Each voxel is represented by a five-dimensional vector, in which the time property is not involved. Inspired by Ref. [13], the time property of each voxel is modeled in an implicit fashion such that data points are organized in subspaces: one color subspace and two spatial subspaces (each frame has one spatial subspace). Each supervoxel is composed of two superpixels, each of which is associated to a Gaussian distribution with unknown parameters. To estimate the parameters, voxels are assumed to be observed from a mixture of Gaussian distributions. Based on maximum likelihood estimation, the unknown parameters are estimated using the expectation–maximization (EM) method. Once the values of the parameters are obtained, each voxel’s supervoxel label is determined to be the one that has the maximum posterior probability.

### 3.1. Problem Formulation

For a given sequence of frames, we use $z_{i}^{t}$ to denote pixel *i* in frame *t*, with i∈I and t∈T , where I is the pixel index set and *T* is the frame set. The symbol |·| denotes the number of elements in a given set; e.g., |I| is the number of pixels in each frame, and |T| is the number of frames. The width and height of the frames are denoted by *W* and *H*, respectively. Hence, we have |I|=W·H .

**Figure 3:**
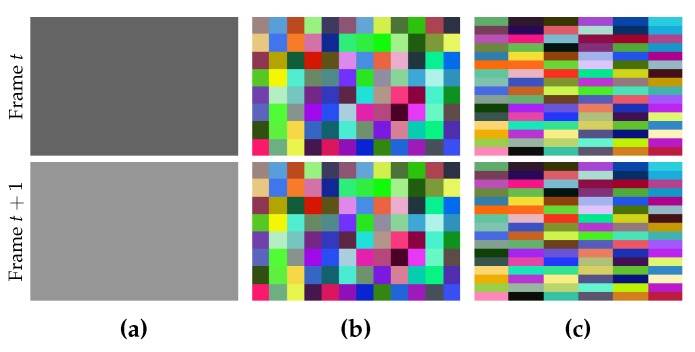
Supervoxel segmentation with different values for $v_{x}$ and $v_{y}$ . (**a**) two successive synthetic frames with constant colors; (**b**) supervoxels with $v_{x}=v_{y}$ ; (**c**) supervoxels with $v_{x}=4v_{y}$ ; In both (**b**) and (**c**), voxels with the same color form a supervoxel. Although (**b**) and (**c**) use different $v_{x}$ and $v_{y}$ , the number of the generated supervoxels are the same, but the shapes are very different.

The desired size of each superpixel in a given frame is specified by $v_{x}\cdot v_{y}$ , where $v_{x}$ and $v_{y}$ are the number of pixels along width and height, respectively. Usually, the values of $v_{x}$ and $v_{y}$ are given by users. If we use values with a large difference, the shape of the generated superpixels in each frame will tend to be a narrow rectangle (see Figure 3). Although people can assign different values to them, it is encouraged to assign the same value, or at least two different values with a very small difference, unless the narrow shape is useful for a special purpose.

Supervoxel segmentation is to assign each voxel a unique label. Voxels with the same label form a supervoxel. All the possible supervoxel labels form a supervoxel set K={0,1,…,K−1} , where K=|K| is the number of supervoxels, which is computed in Equation (1):

(1) $$n_{x}=\frac{W}{v_{x}}\;,n_{y}=\frac{H}{v_{y}}\;,K=n_{x}\cdot n_{y}\;,$$

where $n_{x}$ and $n_{y}$ are the desired numbers of superpixels along width and height for a single frame. For simplicity, we assume $W\;\mathrm{mod}\;v_{x}=0$ and $H\;\mathrm{mod}\;v_{y}=0$ .

In this work, the supervoxel segmentation procedure is performed on every two frames. Therefore, a supervoxel is only valid on two successive frames. For instance, if some voxels in frame *t* and frame t+1 share the same supervoxel label, they are a subset of the same supervoxel. However, if the voxels are in frame *t* and frame t+3 , they are not in the same supervoxel. In order to provide cues for some subsequent applications (e.g., object tracking), frame *t*—where t≠0 and t≠|T|−1 —will be used two times. In the first time, the segmentation is performed on frame t−1 and frame *t*. In the second time, the same procedure of segmentation is performed on frames *t* and t+1 . By doing this, frame *t* will have two valid superpixel segmentations. The detected regions in frame *t* can be propagated by finding the overlapping superpixels in the second segmentation (see Figure 2 for an illustration). Because the same methods are used to segment any two frames, the supervoxel problem becomes finding *K* supervoxels for frame *t* and t+1 such that the generated supervoxels are similar in size, adhere to object boundaries well, and are temporally consistent. Therefore, only frame *t* and frame t+1 will be considered in Section 3.2.

### 3.2. The Model

To distinguish variables between two frames, a symbol with a hat at its top indicates that the symbol is related to frame t+1 . Each voxel in frame *t* is represented by a five-dimensional vector including spatial location $\left(x_{i}\;,y_{i}\right)$ and three CIELAB color components—lightness $u_{i}$ and two color components $a_{i}$ and $b_{i}$ . This can be expressed by

(2) $$z_{i}={\left(x_{i},y_{i},u_{i},a_{i},b_{i}\right)}^{T}\;,$$

in which the superscript *T* indicates vector transpose. Similarly, voxel *i* in frame t+1 is represented using


(3) $${\hat{z}}_{i}={\left({\hat{x}}_{i},{\hat{y}}_{i},{\hat{u}}_{i},{\hat{a}}_{i},{\hat{b}}_{i}\right)}^{T}\;.$$

For two given frames *t* and t+1 , voxels are assumed to be distributed according to mixtures of Gaussian distributions in which each Gaussian distribution corresponds to a superpixel. Gaussian function g(·;·) is defined in Equation (4), where the semicolon is used to separate variables and parameters:

(4) $$g\left(z;\mu ,\Sigma \right)=\frac{1}{\sqrt[{D}]{2\pi }\sqrt{\det(\Sigma )}}\exp\left\{{\left(z-\mu \right)}^{T}\;\Sigma^{-1}\;\left(z-\mu \right)\right\}\;,$$

in which *z* is a *D*-dimensional column vector, μ and Σ are mean vector and covariance matrix, respectively.

Generally, if voxels in different frames have similar colors and have small spatial distances, they generally belong to the same object. This is particularly true in the videos with moving objects. To incorporate this notion into our model, we use different parameters for spatial information but the same parameters for color information for the same supervoxel in the definition of voxel density functions d(·) and $\hat{d}\left(\cdot \right)$ , as shown in Equation (5). This is the key point to ensure temporal consistency:

(5) $$d\left(z_{i}\right)=\sum_{k\in K_{i}}P_{k}^{i}\cdot g\left(z_{i};\mu_{k},\Sigma_{k}\right)\;,\;\hat{d}\left({\hat{z}}_{i}\right)=\sum_{k\in K_{i}}P_{k}^{i}\cdot g\left({\hat{z}}_{i};{\hat{\mu }}_{k},{\hat{\Sigma }}_{k}\right)\;,$$

in which

(6) $$\mu_{k}={\left(\mu_{k}^{s},\mu_{k}^{c}\right)}^{T}\;,\;{\hat{\mu }}_{k}={\left({\hat{\mu }}_{k}^{s},\mu_{k}^{c}\right)}^{T}\;,\;\Sigma_{k}=\left[\begin{array}{cc} \Sigma_{k}^{s} & 0\\ 0 & \Sigma_{k}^{c} \end{array}\right]\;,\;{\hat{\Sigma }}_{k}=\left[\begin{array}{cc} {\hat{\Sigma }}_{k}^{s} & 0\\ 0 & \Sigma_{k}^{c} \end{array}\right]\;,$$

where $\mu_{k}^{s}$ and $\Sigma_{k}^{s}$ are spatial mean vectors and spatial covariance matrices for frame *t* with k∈K . Their parallel notations ${\hat{\mu }}_{k}^{s}$ and ${\hat{\Sigma }}_{k}^{s}$ are for frame t+1 . The color mean vectors $\mu_{k}^{c}$ and color covariance matrices $\Sigma_{k}^{c}$ are for both frame *t* and frame t+1 . Supervoxel *k* can be characterized by the parameters $\mu_{k}^{s}$ , ${\hat{\mu }}_{k}^{s}$ , $\Sigma_{k}^{s}$ , ${\hat{\Sigma }}_{k}^{s}$ , $\mu_{k}^{c}$ , and $\Sigma_{k}^{c}$ , and thus each supervoxel corresponds to two Gaussian distributions. Accounting for the locality of supervoxels, $K_{i}$ in Equation (5) is a subset of K , and its elements are related to the spatial location of voxel *i*. The definition of $K_{i}$ will be discussed later. Instead of defining $P_{k}^{i}$ as a variable just like existing Gaussian mixture models, $P_{k}^{i}$ is defined as a constant $1/|K_{i}|$ here to make the generated supervoxels similar in size.

Once two successive frames *t* and t+1 are given, parameters in the Gaussian densities can be inferred and the label of each voxel, $l_{i}$ for frame *t* and ${\hat{l}}_{i}$ for frame t+1 , will be determined by the following equations:

(7) $$l_{i}=\arg_{k\in K_{i}}\max\mathrm{Pr}\left(k\;|\;z_{i}\right)\;,\;{\hat{l}}_{i}=\arg_{k\in K_{i}}\max\mathrm{Pr}\left(k\;|\;{\hat{z}}_{i}\right)\;,$$

in which $\mathrm{Pr}(k\;|\;z_{i})$ is the probability of assigning voxel *i* in frame *t* to supervoxel *k* given the observation $z_{i}$ . $\mathrm{Pr}(k\;|\;{\hat{z}}_{i})$ has a similar meaning. By applying Bayes rule, the posterior probabilities in Equation (7) can be expressed by

(8) $$\mathrm{Pr}\left(k\;|\;z_{i}\right)=\frac{g\left(z_{i};\mu_{k},\Sigma_{k}\right)\;P_{k}^{i}}{p(z_{i})}\;,\;\mathrm{Pr}\left(k\;|\;{\hat{z}}_{i}\right)=\frac{g\left({\hat{z}}_{i};{\hat{\mu }}_{k},{\hat{\Sigma }}_{k}\right)\;P_{k}^{i}}{p({\hat{z}}_{i})}\;.$$

Based on Equations (7) and (8), $l_{i}$ and ${\hat{l}}_{i}$ can be computed by the equivalent equations, as shown below:

(9) $$l_{i}=\arg_{k\in K_{i}}\max g\left(z_{i};\mu_{k},\Sigma_{k}\right)\;,\;{\hat{l}}_{i}=\arg_{k\in K_{i}}\max g\left({\hat{z}}_{i};{\hat{\mu }}_{k},{\hat{\Sigma }}_{k}\right)\;.$$

### 3.3. Estimating Parameters of Gaussian Distributions

Given two frames *t* and t+1 , we use the method of maximum likelihood to estimate the unknown parameters in the Gaussian distributions. Since the proposed density functions for voxels may be not identical because the elements in $K_{i}$ may be different for different i∈I (see Section 3.4 for details), updating formulas for traditional GMM cannot be simply copied to our new model. Therefore, we will derive the updating formulas for the proposed model in this section by applying the classical expectation–maximization (EM) method to iteratively improve the log-likelihood.

As the voxels in frames *t* and t+1 are assumed to be distributed independently, the log-likelihood $\tilde{L}\left(\theta \right)$ for the two frames can be written out as follows:

(10)L˜(θ)=∑i∈Ilogd(zi)+log(d^(z^i))(11)=∑i∈Ilog1|Ki|2+log∑k∈Kig(zi;θk)+log∑k∈Kig(z^i;θ^k),

where θ is a vector of all the unknown parameters composed of $\mu_{k}^{s}$ , ${\hat{\mu }}_{k}^{s}$ , $\Sigma_{k}^{s}$ , ${\hat{\Sigma }}_{k}^{s}$ , $\mu_{k}^{c}$ , and $\Sigma_{k}^{c}$ with k∈K . For each supervoxel *k*, $\theta_{k}=\left(\mu_{k},\Sigma_{k}\right)$ and ${\hat{\theta }}_{k}=\left({\hat{\mu }}_{k},{\hat{\Sigma }}_{k}\right)$ . Because the number of elements in supervoxel set $K_{i}$ is constant (see Section 3.4), the value of the parameter θ that maximizes $\tilde{L}\left(\theta \right)$ is equal to the value that maximizes the following function L(θ) :

(12) $$L\left(\theta \right)=\sum_{i\in I}\left\{\log\left(\sum_{k\in K_{i}}g\left(z_{i};\theta_{k}\right)\right)+\log\left(\sum_{k\in K_{i}}g\left({\hat{z}}_{i};{\hat{\theta }}_{k}\right)\right)\right\}\;.$$

It is difficult to find the optimal value for θ by maximizing L(θ) directly. We insert new variables $R_{k}^{i}$ and ${\hat{R}}_{k}^{i}$ into Equation (12) such that


(13) $$\sum_{k\in K_{i}}R_{k}^{i}=1\;,\;R_{k}^{i}\geq 0\;,\;\sum_{k\in K_{i}}{\hat{R}}_{k}^{i}=1\;,\;{\hat{R}}_{k}^{i}\geq 0\;,\;i\in I\;,\;k\in K_{i}\;.$$

Then, Equation (12) will become Equation (14). By applying Jensen’s inequality, the EM method is to alternatively find $R=\{R_{k}^{i},{\hat{R}}_{k}^{i}|i\in I,k\in K_{i}\}$ satisfying the equality of the inequality in Equation (15) with parameters in θ being known (expectation step or E-step), and find parameters in θ that maximize Q(R,θ), which is defined in Equation (15) using the obtained *R* (maximization step or M-step):

(14)L(θ)=∑i∈Ilog∑k∈KiRkig(zi;θk)Rki+log∑k∈KiR^kig(z^i;θ^k)R^ki(15)≥∑i∈I∑k∈KiRkilogg(zi;θk)Rki+∑k∈KiR^kilogg(z^i;θ^k)R^ki≐Q(R,θ).

E-step: According to the theory of Jensen’s inequality, equality holds if and only if

(16) $$\frac{g(z_{i};\theta_{k})}{R_{k}^{i}}≐\alpha_{i}\;,\;\mathrm{and}\;\frac{g({\hat{z}}_{i};{\hat{\theta }}_{k})}{{\hat{R}}_{k}^{i}}≐{\hat{\alpha }}_{i}$$

are constant. With the constraints in Equation (13), formulas to update *R* can be derived by eliminating the temporal variables $\alpha_{i}$ and ${\hat{\alpha }}_{i}$ in Equation (16), as shown below:

(17) $$R_{k}^{i}=\frac{g(z_{i};\theta_{k})}{\sum_{k\in K_{i}}g\left(z_{i};\theta_{k}\right)}\;,\;{\hat{R}}_{k}^{i}=\frac{g({\hat{z}}_{i};{\hat{\theta }}_{k})}{\sum_{k\in K_{i}}g\left({\hat{z}}_{i};{\hat{\theta }}_{k}\right)}\;,$$

where $K_{i}$ is defined in Section 3.4.

M-step: To find the parameters θ that maximize Q(R,θ) , we first get the partial derivatives of Q(R,θ) with respect to different components of θ , as shown in Equations (18)–(22), and then set them to zero to get the optimal θ , which is shown in Equations (24)–(27):

(18) $$\frac{\partial Q(R,\theta )}{\partial \mu_{k}^{s}}=\sum_{i\in I_{k}}\left\{R_{k}^{i}{\left(\Sigma_{k}^{s}\right)}^{-1}\left(z_{i}^{s}-\mu_{k}^{s}\right)\right\}\;,\;\frac{\partial Q(R,\theta )}{\partial {\hat{\mu }}_{k}^{s}}=\sum_{i\in I_{k}}\left\{{\hat{R}}_{k}^{i}{\left({\hat{\Sigma }}_{k}^{s}\right)}^{-1}\left({\hat{z}}_{i}^{s}-{\hat{\mu }}_{k}^{s}\right)\right\}\;,$$

(19) $$\begin{array}{cc} \frac{\partial Q(R,\theta )}{\partial \Sigma_{k}^{s}}= & \sum_{i\in I_{k}}\frac{R_{k}^{i}}{2}\left\{{\left(\Sigma_{k}^{s}\right)}^{-1}\left(z_{i}^{s}-\mu_{k}^{s}\right){\left(z_{i}^{s}-\mu_{k}^{s}\right)}^{T}{\left(\Sigma_{k}^{s}\right)}^{-1}-{\left(\Sigma_{k}^{s}\right)}^{-1}\right\}\;, \end{array}$$

(20) $$\begin{array}{cc} \frac{\partial Q(R,\theta )}{\partial {\hat{\Sigma }}_{k}^{s}}= & \sum_{i\in I_{k}}\frac{{\hat{R}}_{k}^{i}}{2}\left\{{\left({\hat{\Sigma }}_{k}^{s}\right)}^{-1}\left({\hat{z}}_{i}^{s}-{\hat{\mu }}_{k}^{s}\right){\left({\hat{z}}_{i}^{s}-{\hat{\mu }}_{k}^{s}\right)}^{T}{\left({\hat{\Sigma }}_{k}^{s}\right)}^{-1}-{\left({\hat{\Sigma }}_{k}^{s}\right)}^{-1}\right\}\;, \end{array}$$

(21) $$\frac{\partial Q(R,\theta )}{\partial \mu_{k}^{c}}=\sum_{i\in I_{k}}\left\{R_{k}^{i}{\left(\Sigma_{k}^{c}\right)}^{-1}\left(z_{i}^{c}-\mu_{k}^{c}\right)+{\hat{R}}_{k}^{i}{\left(\Sigma_{k}^{c}\right)}^{-1}\left({\hat{z}}_{i}^{c}-\mu_{k}^{c}\right)\right\}\;,$$

(22) $$\begin{array}{cc} \frac{\partial Q(R,\theta )}{\partial \Sigma_{k}^{c}}= & \sum_{i\in I_{k}}\{\frac{R_{k}^{i}}{2}\left\{{\left(\Sigma_{k}^{c}\right)}^{-1}\left(z_{i}^{c}-\mu_{k}^{c}\right){\left(z_{i}^{c}-\mu_{k}^{c}\right)}^{T}{\left(\Sigma_{k}^{c}\right)}^{-1}-{\left(\Sigma_{k}^{c}\right)}^{-1}\right\}+\\ & \frac{{\hat{R}}_{k}^{i}}{2}\left\{{\left(\Sigma_{k}^{c}\right)}^{-1}\left({\hat{z}}_{i}^{c}-\mu_{k}^{c}\right){\left({\hat{z}}_{i}^{c}-\mu_{k}^{c}\right)}^{T}{\left(\Sigma_{k}^{c}\right)}^{-1}-{\left(\Sigma_{k}^{c}\right)}^{-1}\right\}\}\;, \end{array}$$

where $z_{i}^{s}={\left(x_{i},y_{i}\right)}^{T}$ and ${\hat{z}}_{i}^{s}={\left({\hat{x}}_{i},{\hat{y}}_{i}\right)}^{T}$ are spatial vectors of voxel *i* in frame *t* and t+1 , respectively. Similarly, $z_{i}^{c}={\left(u_{i},a_{i},b_{i}\right)}^{T}$ and ${\hat{z}}_{i}^{c}={\left({\hat{u}}_{i},{\hat{a}}_{i},{\hat{b}}_{i}\right)}^{T}$ are color vectors. For each supervoxel *k*, $I_{k}$ is a voxel set that supervoxel *k* may cover, and is deduced from $K_{i}$ as shown in Equation (23):

(23) $$I_{k}=\left\{i\;|\;i\in I\;,k\in K_{i}\right\}\;,$$

(24) $$\begin{array}{cc} \mu_{k}^{s}=\frac{\sum_{i\in I_{k}}R_{k}^{i}z_{i}^{s}}{\sum_{i\in I_{k}}R_{k}^{i}}\;,\;\; & \Sigma_{k}^{s}=\frac{\sum_{i\in I_{k}}R_{k}^{i}\left(z_{i}^{s}-\mu_{k}^{s}\right){\left(z_{i}^{s}-\mu_{k}^{s}\right)}^{T}}{\sum_{i\in I_{k}}R_{k}^{i}}\;, \end{array}$$

(25) $$\begin{array}{cc} {\hat{\mu }}_{k}^{s}=\frac{\sum_{i\in I_{k}}{\hat{R}}_{k}^{i}{\hat{z}}_{i}^{s}}{\sum_{i\in I_{k}}{\hat{R}}_{k}^{i}}\;,\;\; & {\hat{\Sigma }}_{k}^{s}=\frac{\sum_{i\in I_{k}}{\hat{R}}_{k}^{i}\left({\hat{z}}_{i}^{s}-{\hat{\mu }}_{k}^{s}\right){\left({\hat{z}}_{i}^{s}-{\hat{\mu }}_{k}^{s}\right)}^{T}}{\sum_{i\in I_{k}}{\hat{R}}_{k}^{i}}\;, \end{array}$$

(26) $$\mu_{k}^{c}=\frac{\left(R_{k}^{i}z_{i}^{c}+{\hat{R}}_{k}^{i}{\hat{z}}_{i}^{c}\right)}{\sum_{i\in I_{k}}(R_{k}^{i}+{\hat{R}}_{k}^{i})}\;,$$

(27) $$\Sigma_{k}^{c}=\frac{R_{k}^{i}\left(z_{i}^{c}-\mu_{k}^{c}\right){\left(z_{i}^{c}-\mu_{k}^{c}\right)}^{T}+{\hat{R}}_{k}^{i}\left({\hat{z}}_{i}^{c}-\mu_{k}^{c}\right){\left({\hat{z}}_{i}^{c}-\mu_{k}^{c}\right)}^{T}}{\sum_{i\in I_{k}}(R_{k}^{i}+{\hat{R}}_{k}^{i})}\;.$$

Usually, the EM method starts by feeding it with a guess of the parameters in θ . Then, *R* and θ will be alternatively updated using the formulas mentioned in E-step and M-step. However, there is a risk that the covariance matrices may become singular and we may fail to obtain their inverse matrices. For example, when the voxels in $I_{k}$ have the same constant color, during the iteration of EM $\Sigma_{k}^{c}$ will become zero matrix, which is obviously singular. To avoid this trouble, one can perturb each $z_{i}^{c}$ and each ${\hat{z}}_{i}^{c}$ with a small random vector before the EM iterations. This trick may succeed in most cases, but it may fail when an object in a frame is very narrow (e.g., a straight line), in which case certain covariance matrices $\Sigma_{k}^{s}$ or ${\hat{\Sigma }}_{k}^{s}$ may become singular. In order to prevent the covariance matrices from being singular, we first obtain their eigenvalues and impose a lower bound to the eigenvalues to reproduce the covariance matrices. When an eigenvalue is less than the specified lower bound, we assign the eigenvalue to that lower bound. We use $\lambda_{s}$ and $\lambda_{c}$ to denote the lower bound of spatial eigenvalues and color eigenvalues, respectively. Experimentally, we have found that $\lambda_{s}=2$ and $\lambda_{c}=8$ is appropriate to outperform the state-of-the-art algorithms. We will use this setting for the remaining text.

In theory, the iteration of the EM method will not stop until the parameters in θ converge. However, EM needs hundreds of iterations to reach the condition of convergence, resulting in a low computational efficiency. In practice, aiming to reduce run-time, we use a fixed number of iterations M=20 , which is sufficient in practice for generating supervoxels with state-of-the-art accuracy.

### 3.4. Defining $K_{i}$ and Initializing θ

**Figure 4:**
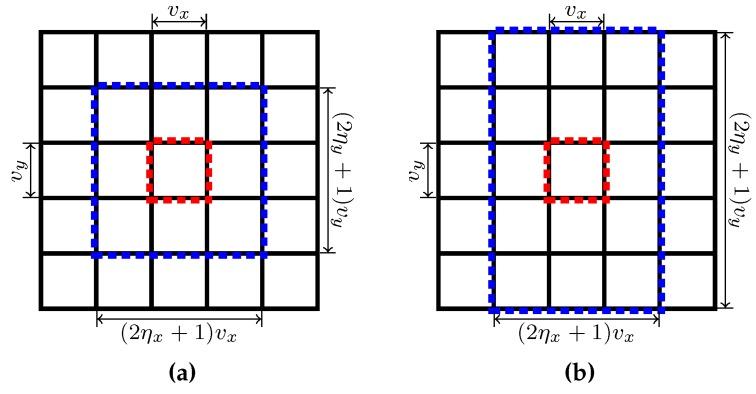
Illustration of anchor region and overlap region with two different settings for $\eta_{x}$ and $\eta_{y}$ : (**a**) $\eta_{x}=\eta_{y}=1$ , (**b**) $\eta_{x}=1$ , $\eta_{y}=2$ . In this example of both (**a**) and (**b**), $n_{x}=n_{y}=5$ and the 25 anchor regions are marked with black rectangles. The region within the blue rectangle is the overlap region $I_{k}$ of supervoxel k=13 whose anchor region is highlighted by a red rectangle.

The definition of supervoxel subsets $K_{i}$ of K will be discussed in this section using mainly the notations mentioned in Section 3.1. After the value of $v_{x}$ and $v_{y}$ are assigned, we define $n_{x}\cdot n_{y}$ rectangle regions called *anchor regions*, with each anchor region corresponding to a supervoxel. As illustrated in Figure 4, for each frame, an anchor region contains $v_{x}\cdot v_{y}$ voxels and all the anchor regions are regularly placed on every frame. For a given supervoxel *k*, all voxels in its anchor region are assigned an *anchor label* $h_{i}=k$ . Then, $K_{i}$ is defined using the anchor label of voxel *i* using the following equations:

(28) $$\begin{array}{c} h_{i}^{x}≐h_{i}\;\mathrm{mod}\;n_{x}\;,\;h_{i}^{y}≐\left\lfloor h_{i}/n_{x}\right\rfloor \;, \end{array}$$

(29) $$\begin{array}{c} K_{i}≐\left\{k_{y}+k_{x}\cdot n_{x}|\begin{array}{c} k_{x}\in \left\{h_{i}^{x}-\eta_{x},\ldots ,h_{i}^{x}+\eta_{x}\right\},0\leq k_{x}\leq n_{x}-1\\ k_{y}\in \left\{h_{i}^{y}-\eta_{y},\ldots ,h_{i}^{y}+\eta_{y}\right\},0\leq k_{y}\leq n_{y}-1 \end{array}\right\}, \end{array}$$

where $\eta_{x}$ and $\eta_{y}$ are parameters used to control the number of supervoxels from which each voxel *i* may be generated. Clearly, at least one of the two parameters must be greater than or equal to 1.

Recall the definition of $I_{k}$ in Equation (23) of Section 3.3. Elements of $I_{k}$ are in turn determined by $K_{i}$ . The voxel set $I_{k}$ is called the *k*-th supervoxel’s *overlap region*, into which voxels in supervoxel *k* may spread. With the help of Figure 4, it is easy to conclude that an overlap region $I_{k}$ of a supervoxel *k* is the region whose center is the anchor region of the supervoxel *k* and whose width and height can be divided evenly by $v_{x}$ and $v_{y}$ respectively, and can be expressed by $\eta_{x}$ and $\eta_{y}$ using $\left(2\eta_{x}+1\right)v_{x}$ and $\left(2\eta_{y}+1\right)v_{y}$ . There are some exceptions, however. When an anchor region *k* is at the boundary of a frame, the size of $I_{k}$ may be less than $\left(2\eta_{x}+1\right)v_{x}\cdot \left(2\eta_{y}+1\right)v_{y}$ , but is at least $\left(\eta_{x}+1\right)v_{x}\cdot \left(\eta_{y}+1\right)v_{y}$ , which is the case in which the anchor region is at one of the four corners of the frame. This conclusion can be used to deduce the computational complexity of our algorithm. As discussed in Section 3.5, the computational complexity can be affected by $\eta_{x}$ and $\eta_{y}$ . A large value for $\eta_{x}\cdot \eta_{y}$ will increase the run-time of the algorithm. Meanwhile, a large value for $\eta_{x}\cdot \eta_{y}$ indicates that a supervoxel has a large overlap region, which may result in a better performance in temporal consistency. By default, we use $\eta_{x}=\eta_{y}=2$ for our experiments.

In our model, a Gaussian distribution represents a superpixel in a single frame, and two Gaussian distributions with the same color parameters (color mean vector and color covariance matrix) but with different spatial parameters represent a single supervoxel. As we expect that the generated superpixels are regularly distributed on each frame, it is straightforward to initialize $\mu_{k}^{s}$ and ${\hat{\mu }}_{k}^{s}$ using the center of the *k*-th anchor region. Since a color mean vector $\mu_{k}^{c}$ varies according to the color information of two frames (see Equation (26)) during the EM iterations, the $\mu_{k}^{c}$ is initialized by the mean color of the two voxels at the center of the *k*-th anchor region of each frame.

For each supervoxel *k*, the corresponding covariance matrices serve as normalizers for the squares of the Euclidean distances (refer to Equations (4) and (9)). To initialize each of the covariance matrices, the idea is to assign their diagonals with the same value, which can be interpreted as a distance within which two voxels tend to be in the same supervoxel. We have found that it is sufficient to initialize color covariance matrices with a color distance $\sigma_{c}=10$ (see Equation (30)) and a small perturbation for $\sigma_{c}$ affects the result less. As we hope each superpixel for a single frame will have the same size $v_{x}\cdot v_{y}$ , the spatial covariance matrices can be initialized using Equation (30):

(30) $$\begin{array}{c} \Sigma_{k}^{c}=\left[\begin{array}{ccc} {\left(\sigma_{c}\right)}^{2} & 0 & 0\\ 0 & {\left(\sigma_{c}\right)}^{2} & 0\\ 0 & 0 & {\left(\sigma_{c}\right)}^{2} \end{array}\right],\Sigma_{k}^{s}={\hat{\Sigma }}_{k}^{s}=\left[\begin{array}{cc} {\left(v_{x}\right)}^{2} & 0\\ 0 & {\left(v_{y}\right)}^{2} \end{array}\right]. \end{array}$$

### 3.5. Computational Complexity

With the discussion above, for any two successive frames, the proposed algorithm can be summarized in Algorithm 1. The proposed algorithm is composed of three major procedures, initializing θ (line 1 to line 3), updating *R* (line 5 to line 9), and updating θ (line 10 to line 13). It is obvious that the initialization of θ needs a computational cost of O(|K|)=O(K) , where *K* is the number of desired supervoxels in two frames and is originally defined in Equation (1).

**Algorithm 1** The proposed supervoxel algorithm.
**Input:**

$v_{x}$
 and 
$v_{y}$
, two successive frames.
**Output:**

$l_{i}$
 and 
${\hat{l}}_{i}$
, 
i∈I
.
    1: **for all**

k∈K

**do**

    2:  Initialize 
$\mu_{k}^{s}$
, 
${\hat{\mu }}_{k}^{s}$
, 
$\Sigma_{k}^{s}$
, 
${\hat{\Sigma }}_{k}^{s}$
, 
$\mu_{k}^{c}$
, 
$\Sigma_{k}^{c}$
 (refer to Section 3.4).
    3: **end for**
    4: **for**

m=1
 to *M*
**do** {refer to Section 3.3 for the value of *M*}
    5:  **for all**

i∈I

**do**
    6:   **for all**

$k\in K_{i}$
**do** {refer to Section 3.4 for 
$K_{i}$
}
    7:    Update 
$R_{k}^{i}$
 and 
${\hat{R}}_{k}^{i}$
 using Equation (17).
    8:   **end for**
    9:  **end for**
  10:  **for all**

k∈K

**do**
  11:   Update 
$\mu_{k}^{s}$
, 
${\hat{\mu }}_{k}^{s}$
, and 
$\mu_{k}^{c}$
 using Equations (24)–(26).
  12:   Update 
$\Sigma_{k}^{s}$
, 
${\hat{\Sigma }}_{k}^{s}$
 and 
$\Sigma_{k}^{c}$
 using Equations (24)–(27).
  13:  **end for**
  14: **end for**
  15: **for all**

i∈I

**do**
  16:  
$l_{i}$
 and 
${\hat{l}}_{i}$
 are determined by Equation (9).
  17: **end for**


According to Equations (28) and (29), the number of elements in $K_{i}$ satisfies the following inequality:

(31) $$\left(\eta_{x}+1\right)\cdot \left(\eta_{y}+1\right)\leq \left|K_{i}\right|\leq \left(2\eta_{x}+1\right)\cdot \left(2\eta_{y}+1\right)\;.$$

For each voxel *i*, updating $R_{k}^{i}$ or ${\hat{R}}_{k}^{i}$ , $k\in K_{i}$ needs time $O(|K_{i}|)$ . For all the elements in *R*, we therefore have a computational complexity $O(\eta_{x}\cdot \eta_{y}\cdot |I|)$ . Based on Equations (24)–(27), for a given supervoxel *k*, updating the parameters in $\theta_{k}$ or ${\hat{\theta }}_{k}$ needs a time of $O(|I_{k}|)$ . By the conclusions about the size of $I_{k}$ in Section 3.4, we know that

(32) $$\left(\eta_{x}+1\right)\left(\eta_{y}+1\right)v_{x}v_{y}\leq \left|I_{k}\right|\leq \left(2\eta_{x}+1\right)\left(2\eta_{y}+1\right)v_{x}v_{y}.$$

Therefore, the computational complexity for updating θ is

(33) $$O(\eta_{x}\eta_{y}v_{x}v_{y}\left|K|\right)=O\left(\eta_{x}\eta_{y}v_{x}v_{y}n_{x}n_{y}\right)=O(\eta_{x}\eta_{y}|I|).$$

Because |K|≪|I| and we use constant values for $\eta_{x}$ and $\eta_{y}$ , the computational complexity of our algorithm is O(|I|) .

When the input video has more than two frames, every internal frame will have two segmentation results: one generated with its previous frame and another generated with its next frame (refer to Figure 2 for a visual illustration). For the entire video sequence, the complexity is O((|T|−1)·|I|)=O(|T|·|I|) , where |T| is the number of frames in the input video and has been mentioned in Section 3.1.

## 4. Experiments

Our method has been designed to produce supervoxels of similar size. To evaluate the performance of our algorithm, it is reasonable to compare the proposed method with algorithms that are also designed to generate supervoxels of similar size. We compared our method with four of these kinds of algorithms, including video SLIC [1] (vSLIC), PARW [17], vSEEDS [19], and TSP [20], whose source codes are publicly available at their respective research websites. We used the default parameters provided by their authors for all the compared methods. Comparisons of some early methods that oversegment video data without considering the property of similar size can be found in the work of [15].

### 4.1. Quantitative Comparisons

We conducted experiments on the Chen dataset [31] and adopted five metrics to evaluate the quality of the supervoxels generated by different algorithms. This dataset contains eight video sequences, and every frame has a ground truth label. Each metric is compared as a function of the average number of superpixels per frame.

**Figure 5:**
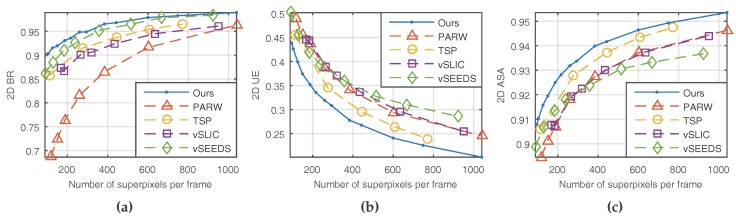
Results of 2D metrics. (**a**) 2D BR; (**b**) 2D UE; (**c**) 2D ASA.

Three of the five metrics are borrowed from still image segmentation, and they are 2D boundary recall (2D BR), 2D under-segmentation error (2D UE), and 2D achievable segmentation accuracy (2D ASA). For a single frame with ground truth labels, 2D BR measures the proportion of ground truth boundaries that fall within two pixels of the superpixel boundaries [3]. As shown in Figure 5a, our method and vSEEDS [19] worked equally well in terms of boundary recall when a relatively large number of superpixels are generated. However, the superiority of our method becomes obvious with the decrease of the number of superpixels.

2D UE, shown in Figure 5b, is another important measure of boundary adherence. For a single frame, given a region $g_{j}$ from the ground truth segmentation and the set of superpixels required to cover it, $\left\{s_{k}|s_{k}\cap g_{j}\neq \phi \right\}$ , where ϕ denotes an empty set, 2D UE measures how many pixels from $s_{k}$ are not in the region $g_{j}$ . Given that |·| is the number of elements in a given set, *G* is the set of ground truth segments, and *M* is the minimum number of pixels in $s_{k}$ overlapping $g_{j}$ , 2D UE can be expressed as

(34) $$2D\;\mathrm{UE}=\frac{1}{\left|I\right|}\left\{\sum_{g_{j}\in G}\left(\sum_{\left\{k\;|\;|s_{k}\cap g_{j}|>M\right\}}\left|s_{k}\right|\right)-\left|I\right|\right\}\;.$$

It is generally accepted to set *M* to five percent of $|s_{k}|$ to account for ambiguities in the ground truth segmentations. Superpixels that do not tightly adhere to the ground truth indicate high 2D UE. Clearly, our method had the minimum under-segmentation error, as shown in Figure 5b.

If we assign every superpixel with the label of a ground truth segment that covers the greatest number of pixels of the corresponding superpixel, 2D ASA measures how much segmentation accuracy we can achieve or how many pixels are correctly segmented, as shown in Equation (35):

(35) $$2D\;\mathrm{ASA}=\frac{1}{\left|I\right|}\sum_{s_{k}}\max\left\{|s_{k}\cap g_{j}|\;|\;g_{j}\in G\right\}\;.$$

Superpixels with high segmentation accuracy will result in a high value of 2D ASA. As shown in Figure 5, our method achieved the best 2D ASA.

**Figure 6:**
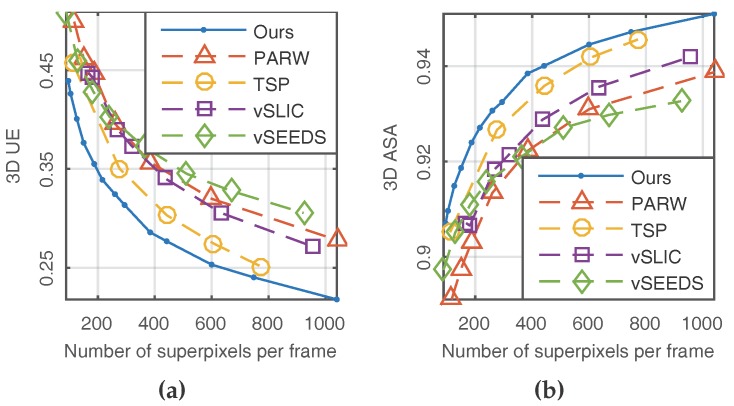
Results of 3D metrics. (**a**) 3D UE; (**b**) 3D ASA.

The other two metrics—namely 3D UE and 3D ASA—were used to evaluate temporal consistency by performing the formulas of 2D UE and 2D ASA on every two consecutive frames. Similarly, low 3D UE and high 3D ASA indicate better performance. As shown in Figure 6, our method presented the best temporal consistency.

**Figure 7:**
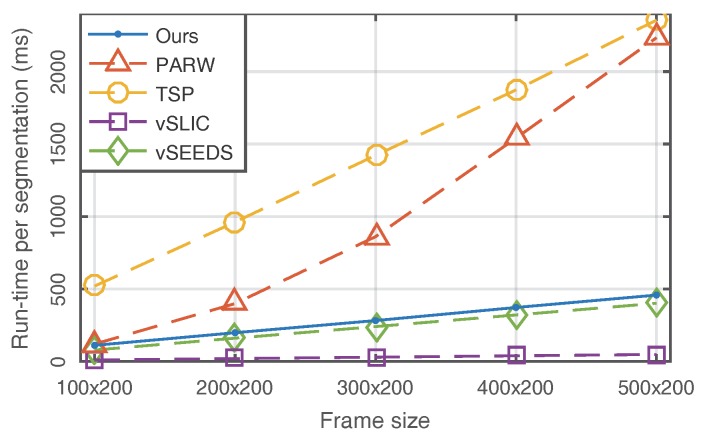
Comparison of run-time.

To compare computational efficiency, we test the selected algorithms on a 4-core Intel CPU at 3.3 GHz. As shown in Figure 7, although our algorithm did not show the best performance in terms of run-time, it is still worth noting that we achieved better results than PARW and TSP and had extremely similar performance to vSEEDS. vSLIC presented the best run-time. However, vSLIC is not a real supervoxel algorithm because it treats video as a fake 3D volume and temporal consistency is not real considered in vSLIC. In addition, Figure 7 experimentally confirms that our method is of linear complexity with respect to frame size.

### 4.2. Qualitative Comparisons

**Figure 8:**
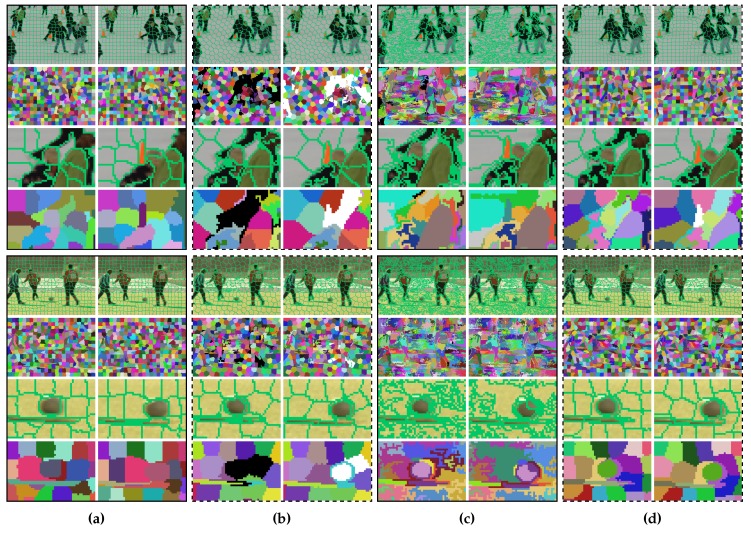
Examples of supervoxel segmentation. (**a**) PARW [17]; (**b**) TSP [20]; (**c**) vSEEDS [19]; (**d**) ours. The algorithms extract approximately the same number of supervoxels. Superpixel boundaries are plotted in the first, third, fifth, and seventh rows. In the remaining rows, we paint voxels of the same supervoxel using the same color. Disappearing and appearing superpixels are painted using black in the first frames and white in the second frames, respectively. The third, fourth, seventh, and eighth rows zoom in on regions of the first, second, fifth, and sixth rows, respectively.

As displayed in Figure 8, we selected four frames from the Chen dataset to compare the segmentation results of four algorithms. Note that vSLIC is not included because it does not consider temporal consistency for real and cannot segment videos with very few frames [1] (e.g., two frames). Our algorithm correctly assigned a new label for the new appearing object, as shown in the fourth row of Figure 8d. Although TSP also correctly detected the new object, this algorithm is easy to overact (certain moving objects are assigned with new labels in Figure 8b). If an object has similar colors in two different frames, our method is able to track it. For instance, most of the supervoxels of our method preserved temporal consistency, as shown in the third last row of Figure 8d, in which the three people and the soccer ball of moving in different directions are similar in color between the two frames. vSEEDS succeeded in segmenting the moving soccer ball, as shown in Figure 8c. However, supervoxels of vSEEDS tend to be considerably dissimilar in size.

**Figure 9:**
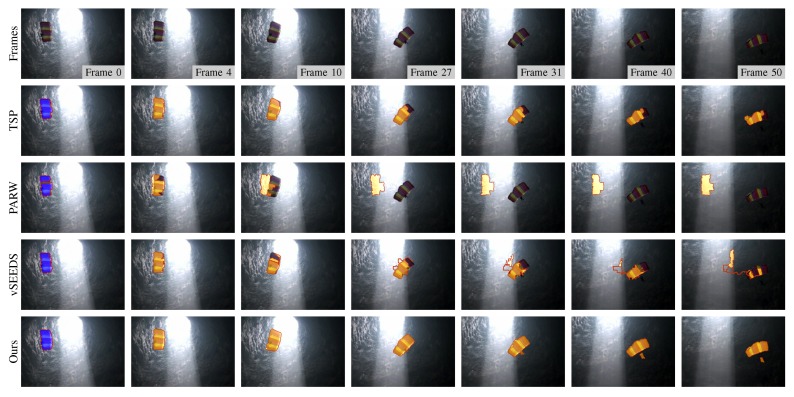
Segmentation results of a moving target that becomes two separated parts during the moving.

**Figure 10:**
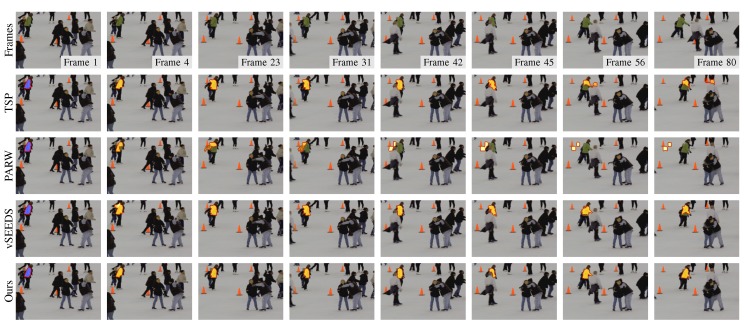
Segmentation results of a moving target that is partially covered by another object during the moving.

**Figure 11:**
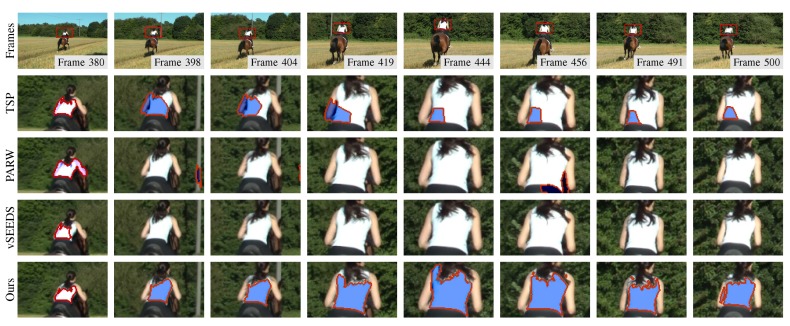
Segmentation results of a moving target of varying size. Only the regions of interest defined by red rectangles in the first row are plotted in the last three rows. This sequence is from [32,33].

Although supervoxel algorithms are not real visual tracking algorithms, supervoxels may be useful in visual tracking. For example, instead of tracking a rectangle window, a tracking algorithm can track the superpixels to either save computing time or improve robustness. In this case, the temporal consistency that supervoxel algorithms attempt to capture becomes an important property that can boost the performance of such tracking algorithms. To compare the performance in terms of temporal consistency when many frames are involved, we manually select some superpixels that cover the same region in one frame and track them using temporal consistency. As shown in Figure 9, Figure 10 and Figure 11, our method presented the best results in tracking the three different kinds of objects.

## 5. Conclusions

A temporal superpixel algorithm was developed based on our novel voxel-related Gaussian mixture model (GMM). Instead of producing immutable superpixels for each frame, we proposed a new scheme for supervoxel segmentation. In this scheme, every two adjacent frames are independently segmented into superpixels and so that every internal frame has two valid superpixel segmentations, which provides our algorithm with a coarse-grained parallel ability and allows subsequent applications to adjust their results on each internal frame to further improve robustness.

In the voxel-related GMM, a supervoxel is represented by two Gaussian distributions, each of which models a superpixel in one frame. To guarantee temporal consistency, the two Gaussian distributions of a supervoxel share the same color parameters. Every superpixel is assumed to be distributed in a local region, resulting in an algorithm with lower complexity than the traditional GMM. According to our experiments, the proposed method outperforms the state-of-the-art algorithms in terms of segmentation accuracy while possessing a competitive computing performance.

As a contribution to open source society, our test code will be publicly available at https://github.com/ahban.
